# Supplementary figures and images for: Early post-infection treatment of SARS-CoV-2 infected macaques with human convalescent plasma with high neutralizing activity had no antiviral effects but moderately reduced lung inflammation
Source: PLoS Pathog. 2022 Apr 20;18(4):e1009925. doi: 10.1371/journal.ppat.1009925 (PMC9060337; doi:10.1371/journal.ppat.1009925)

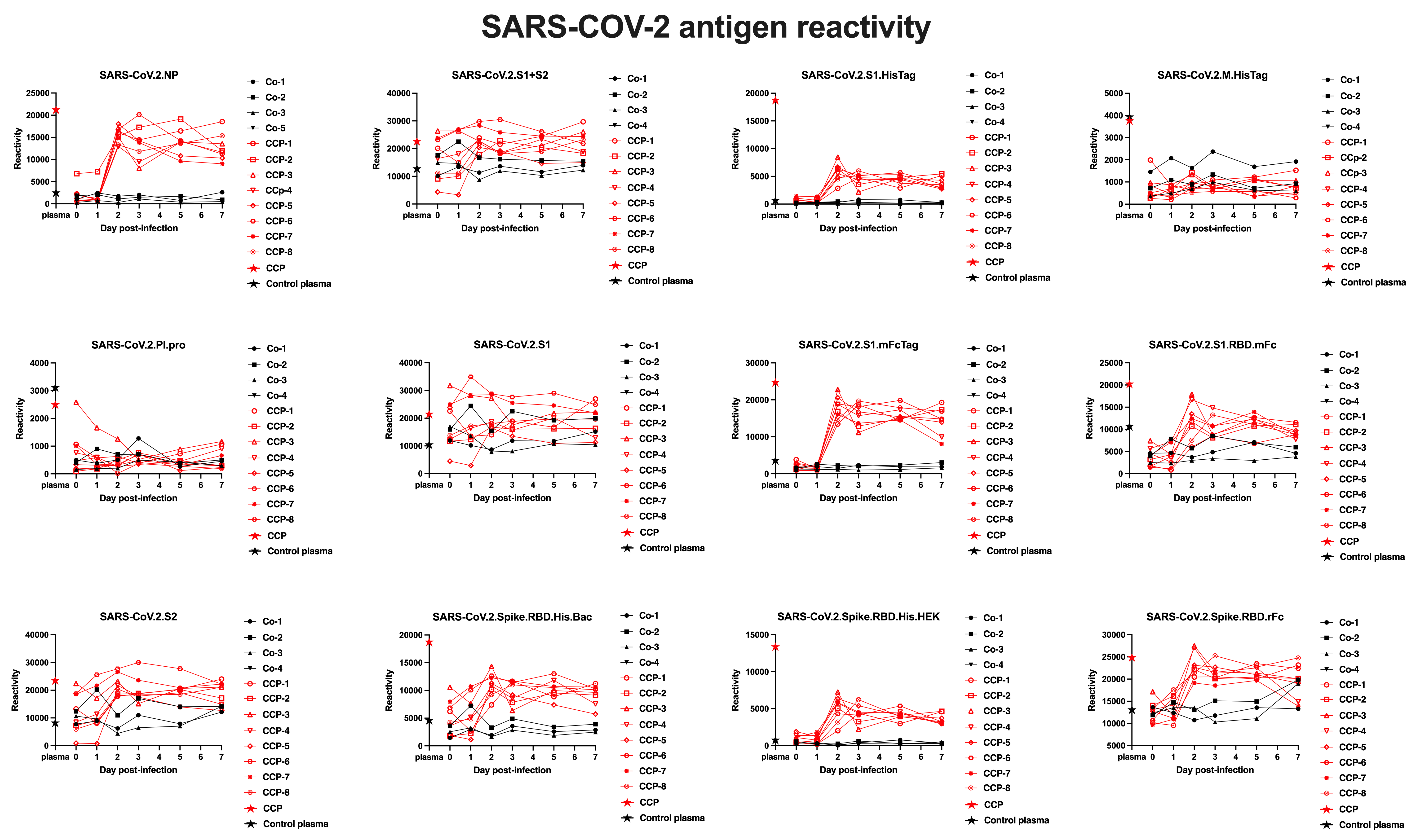

Supplement: S1 Fig — Plasma collected of the macaques before and after infusion with pooled CCP or normal control plasma was tested by coronavirus antigen microarray assay (COVAM). The data on reactivity to SARS-CoV-2 antigens in this assay are represented as individual graphs. The reactivity of the CCP and normal plasma (see Fig 1B) is indicated on the Y-axis as red and black stars, respectively, to demonstrate the dilution effect after transfusion into the macaques. (TIFF) [file ppat.1009925.s001.tiff]

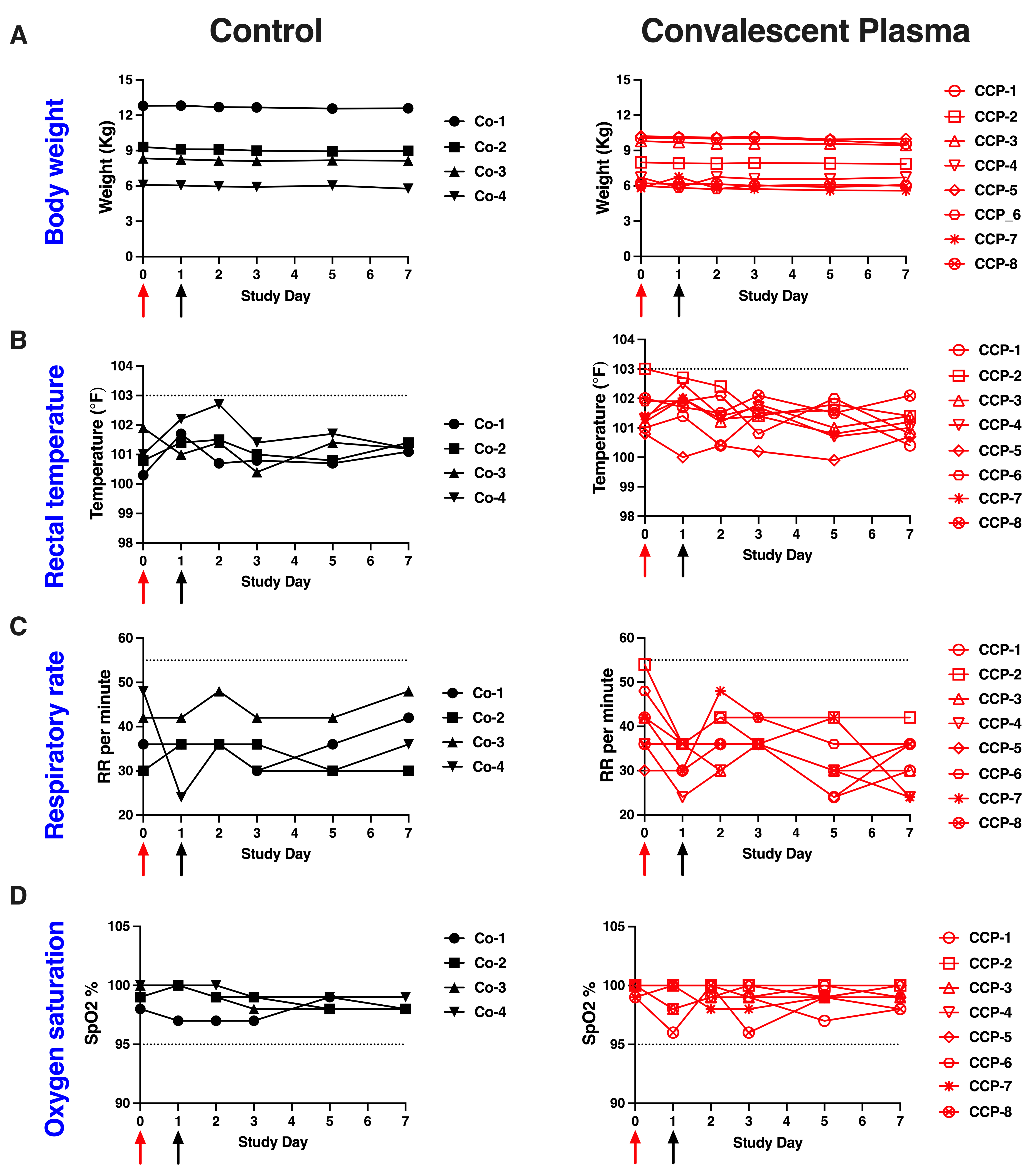

Supplement: S2 Fig — Red and black arrows indicate time of virus inoculation and monoclonal antibody administration on days 0 and 1, respectively. (A) Body weight remained stable. (B) Rectal temperature; horizontal line indicates the cut-off of 103° F, above which ketoprofen treatment was administered. (C) Respiratory rate; the horizontal line indicates a cut-off value of 55 (per minute) as upper normal range. (D) Oxygen saturation measured by pulse oximetry; the horizontal line indicates 95% as the lower end cut-off of the normal range. Total clinical scores, including the markers not graphed above, are presented in Fig 3. (TIFF) [file ppat.1009925.s002.tiff]

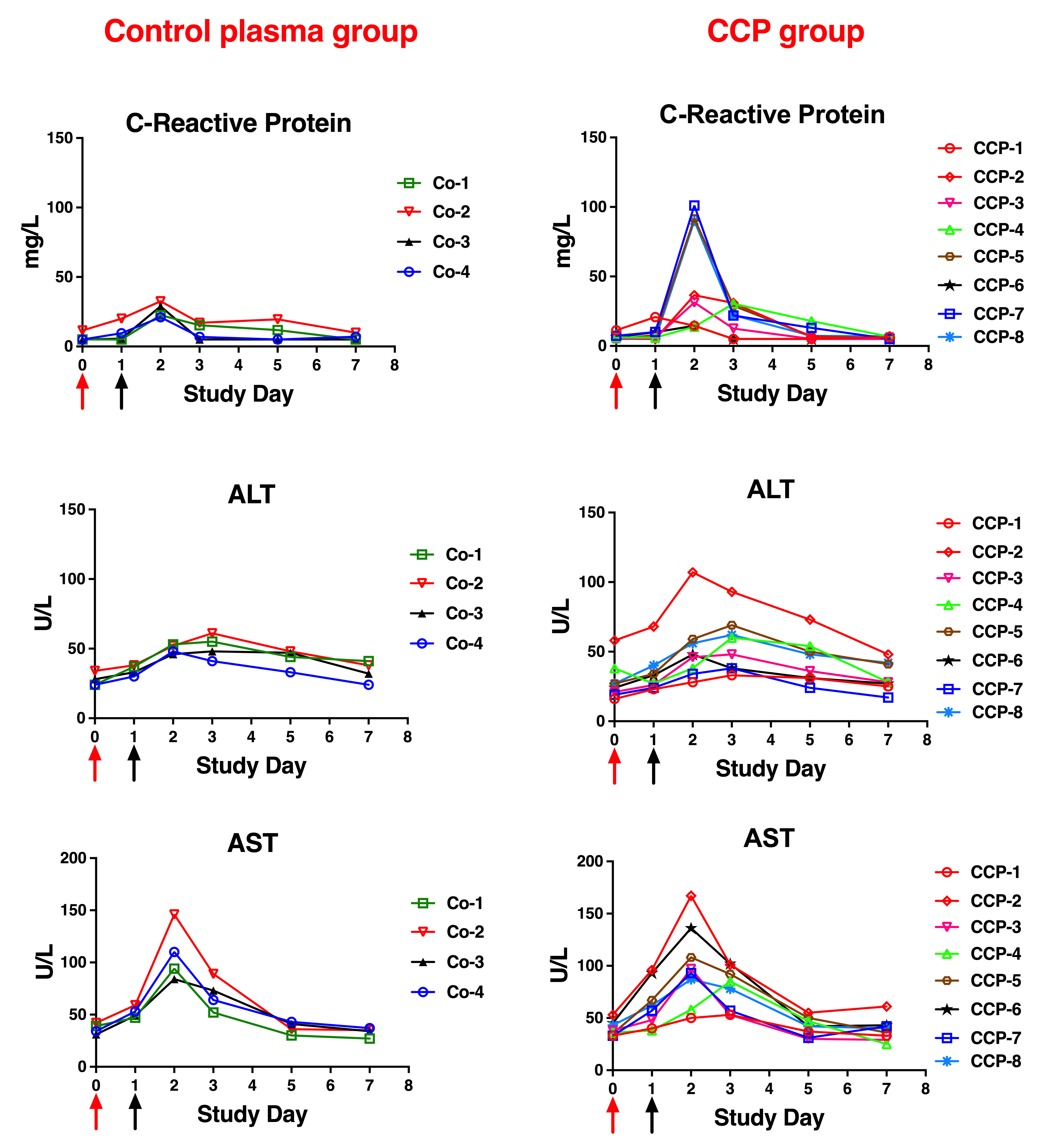

Supplement: S3 Fig — Biochemistry analysis on serum samples was performed using Piccolo® BioChemistry Plus disks. (A) through (C) present C-reactive protein (CRP), alanine aminotransferase (ALT), and aspartate aminotransferase (AST), which showed transient changes during the early stages of infection regardless of the study group. Other markers in the panel did not show any obvious changes. Red and black arrows indicate time of virus inoculation and plasma administration on days 0 and 1, respectively. Using mixed-model analyses, there were no statistically significant differences between the 2 treatment groups after correction for the false discovery rate. (TIFF) [file ppat.1009925.s003.tiff]

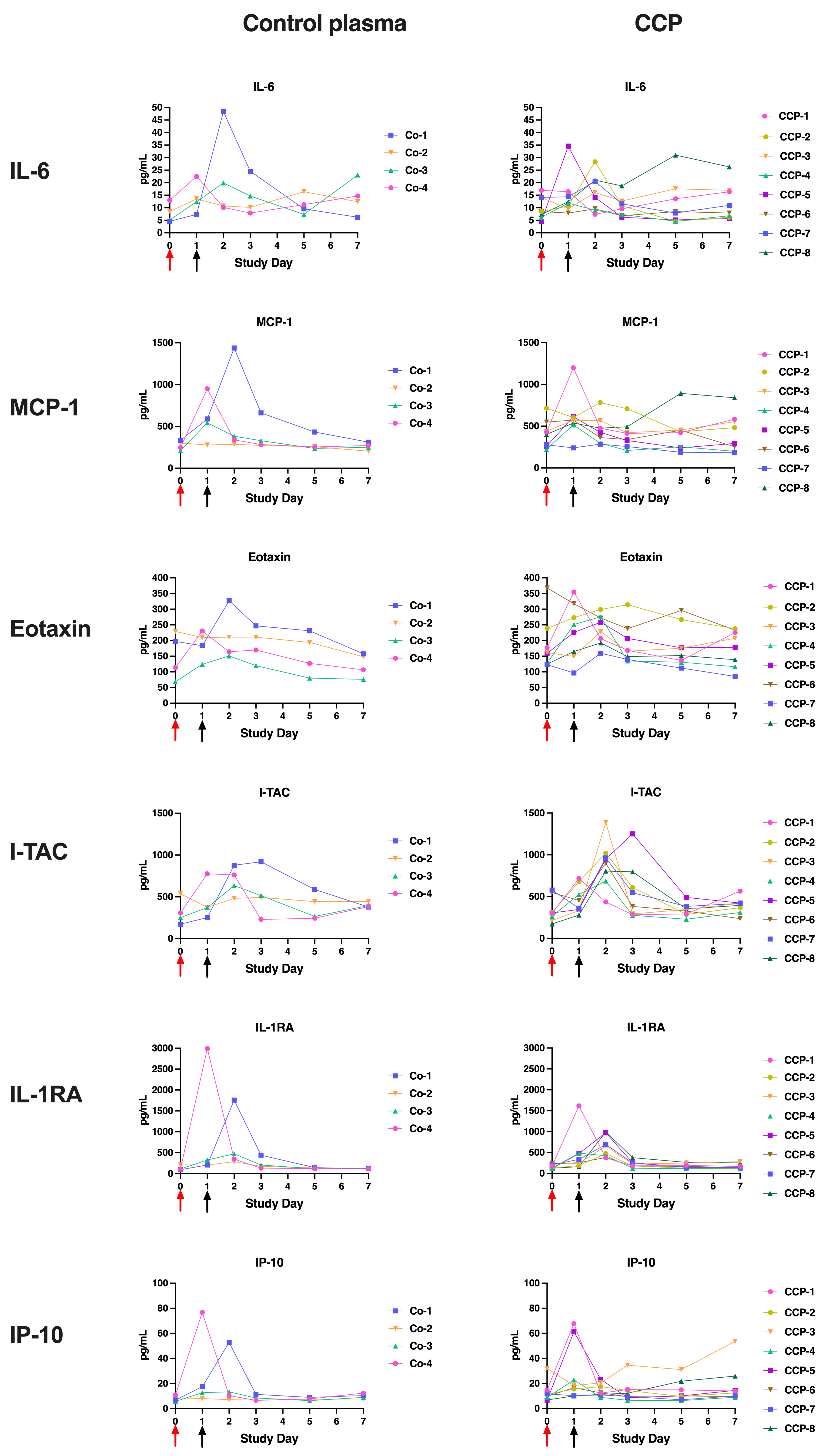

Supplement: S4 Fig — Cytokines and chemokines were measured in plasma using established Luminex-based methodology (see Materials and methods section). Red and black arrows indicate time of virus inoculation and plasma administration on days 0 and 1, respectively. Markers on this figure represent ones that showed the most visible changes after infection. For other markers, see S5 Fig. Using mixed-model analyses, there were no statistically significant differences between the 2 treatment groups after correction for the false discovery rate. (TIFF) [file ppat.1009925.s004.tiff]

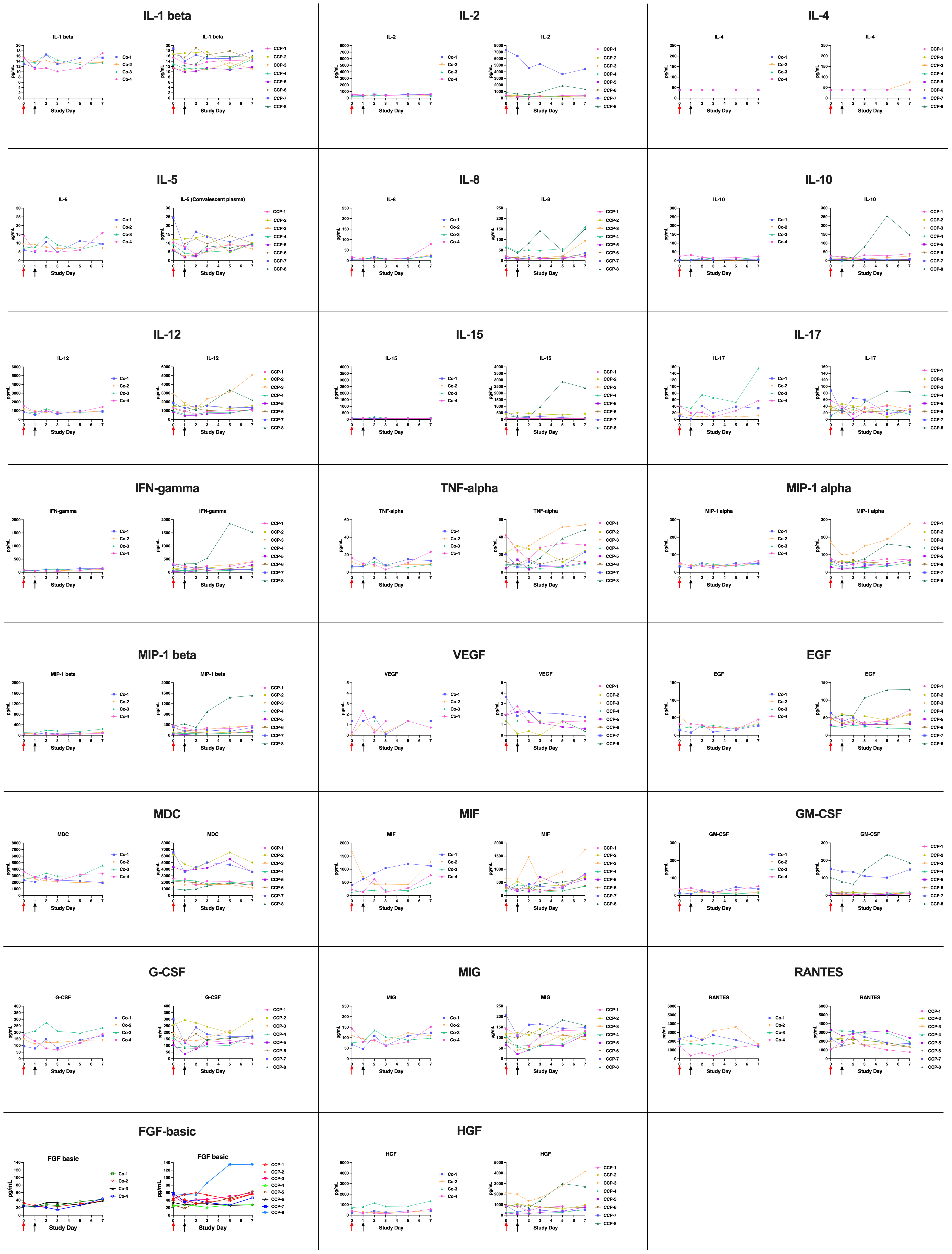

Supplement: S5 Fig — Cytokines and chemokines presented in this panel were ones that did not show consistent changes among animals. The legend is the same as that of S4 Fig. (TIFF) [file ppat.1009925.s005.tiff]

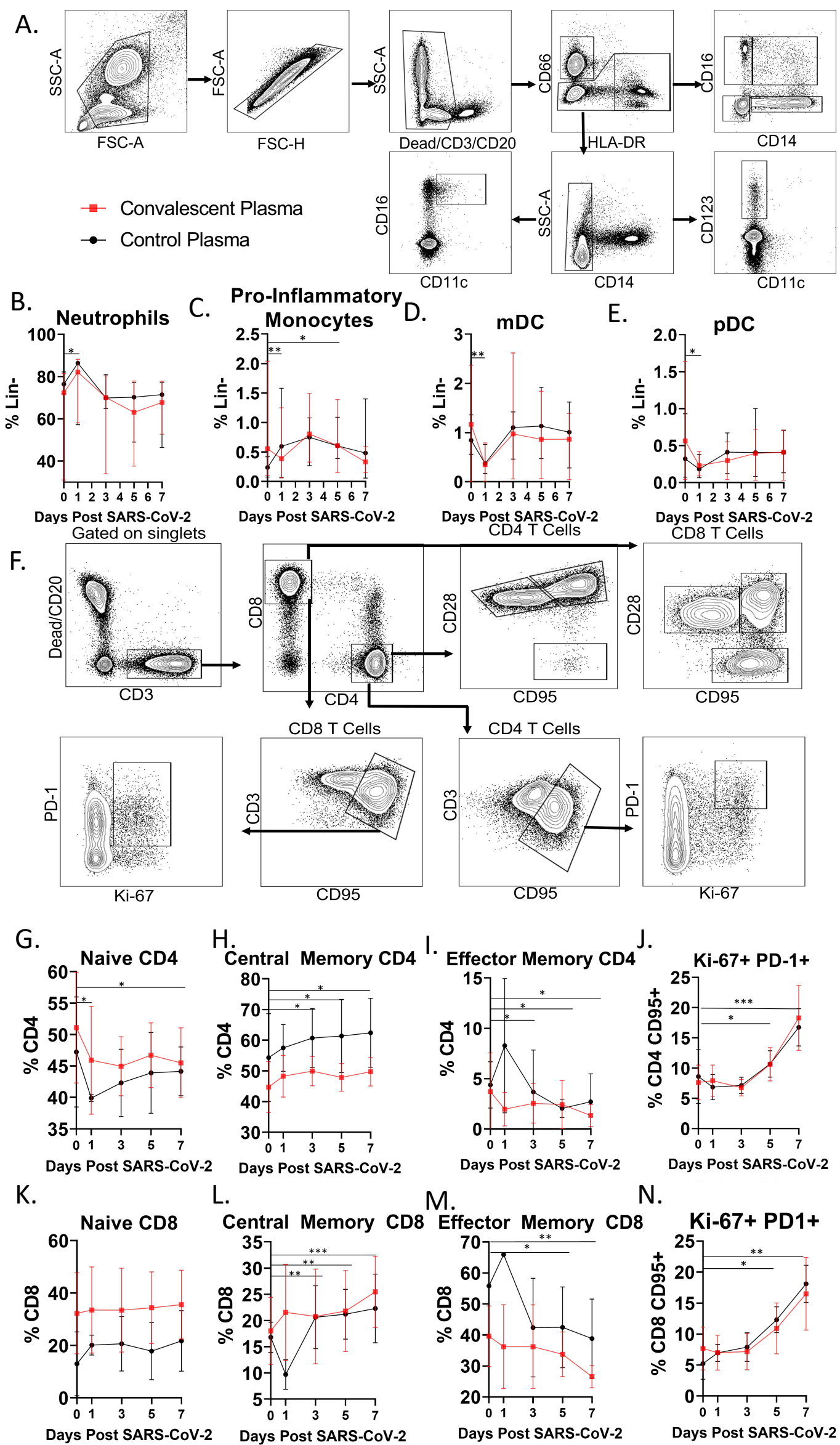

Supplement: S6 Fig — (A) Representative gating strategy for innate immune cells in whole blood. Fluorochromes used: CD66:APC, CD20/CD3/Dead: APC-Cy7, Ki67:AF488, CD14:AF700, CD123:BV421, CD16:BV605, HLA-DR:BV786, CD11c:PE-Cy7. Kinetics of circulating neutrophils, proinflammatory monocytes, mDCs, and pDCs measured at 0,1,3,5, and 7 days post SARS-CoV-2 infection. (B) Representative T cell gating strategy from whole blood. Fluorochromes used: CD25: APC, CD20/Dead: APC-Cy7, Ki67:AF488, CD3:AF700, CD95:BUV737, CD8:BUV805, CD4:BV650, CD69:BV711, CD28:PECF594, PD-1:PE-Cy7. Kinetics of circulating naïve, central memory, effector memory populations, and Ki67+PD-1+ memory cells of CD4 T cell. Kinetics of circulating naïve, central memory, effector memory, and CD69+ effector memory CD8 T cells. Significance was calculated using one tailed paired t test comparing pooled convalescent plasma and normal plasma animals against Day 0 *p = 0.05, **p = 0.01, ***p = 0.001. Statistical analysis yielded no significant different between convalescent and normal plasma groups. (PDF) [file ppat.1009925.s006.pdf]

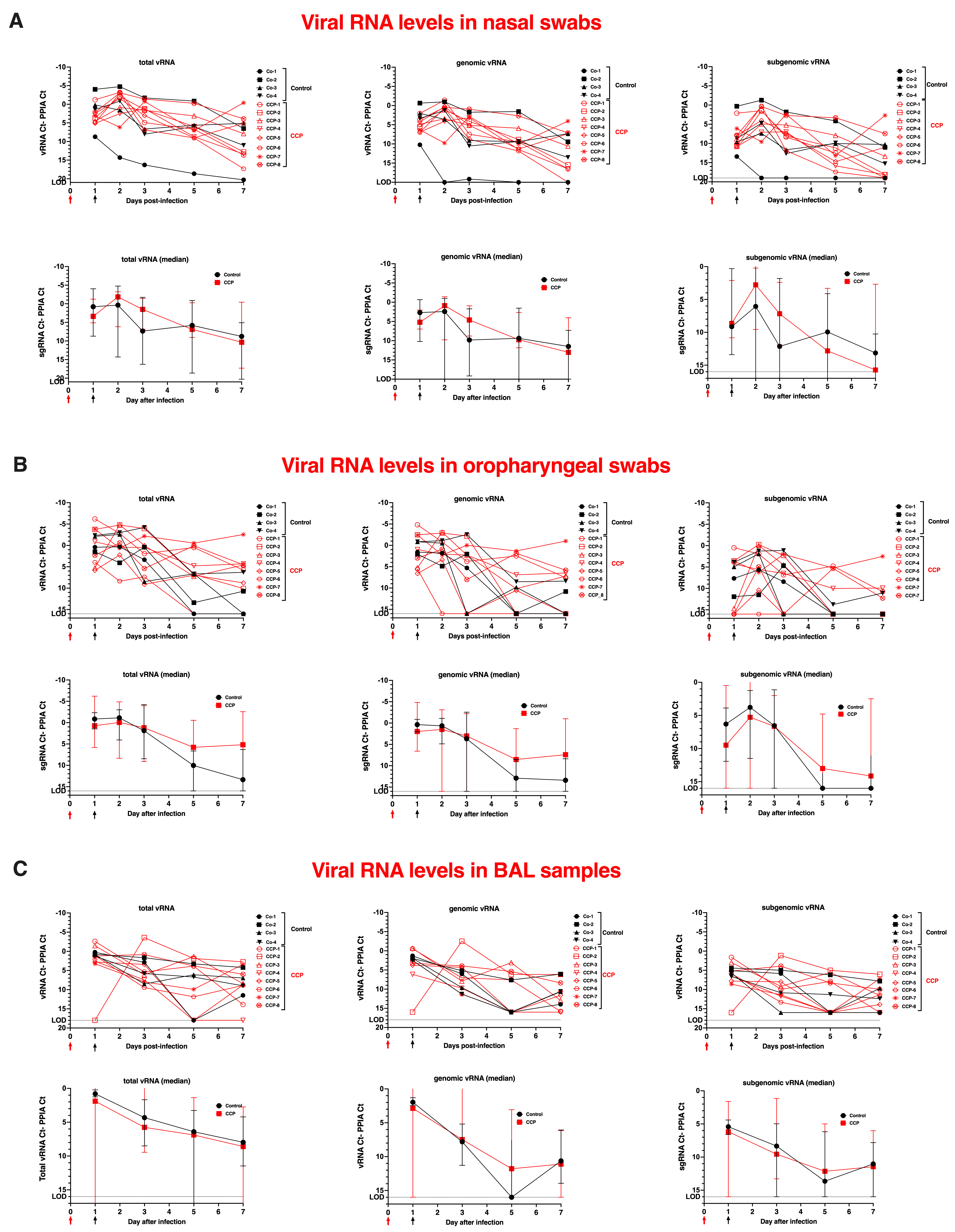

Supplement: S7 Fig — Nasal swabs (A), oropharyngeal swabs (B) and BAL (cell pellets with supernatant) (C) were tested by RT-qPCR for total, genomic and subgenomic viral RNA, and the housekeeping gene PPIA mRNA. Viral RNA levels are expressed relative to PPIA mRNA by graphing the difference in Ct values. For each sample type, the top figures show the individual data (with the intersection of X-axis and Y-axis set near the limit of detection); the bottom figures display the median values per group (with error bars showing the range). Red and black arrows indicate time of virus inoculation and monoclonal antibody administration on days 0 and 1, respectively. (TIFF) [file ppat.1009925.s007.tiff]

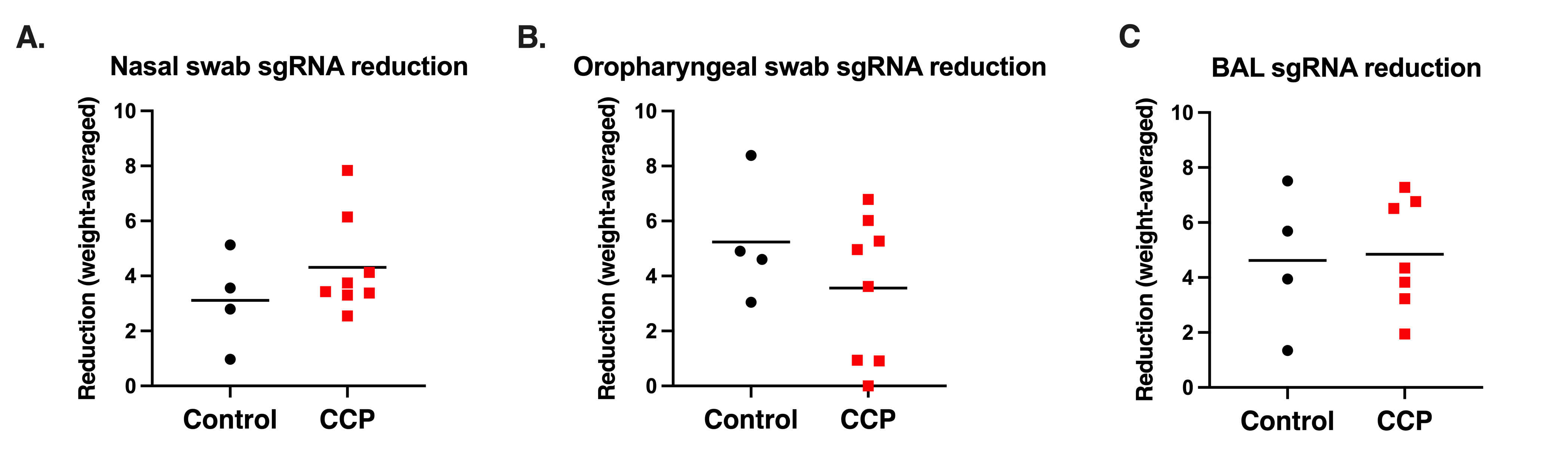

Supplement: S8 Fig — A weighted average analysis was performed on the sgRNA data from nasal and oropharyngeal swabs and BAL (S7 Fig.) to calculate the relative decline of viral RNA (relative to cellular mRNA in the sample) from day 1 to day 7. For each animal, the AUC of relative sgRNA per cellular mRNA over time was tabulated using day 1 as baseline value, and then divided by 6 days to get the weighted average in the decline of sgRNA over the 6-day time period. Lines indicate mean values. On panel C, animal CCP-2 was excluded, as it had no detectable viral RNA in the BAL sample, which precluded this analysis. Statistical analysis revealed no effects between the control and CCP groups (panel A, p = 0.29; panel B: p = 0.30; panel C, p = 0.88; unpaired t-test). (TIFF) [file ppat.1009925.s008.tiff]

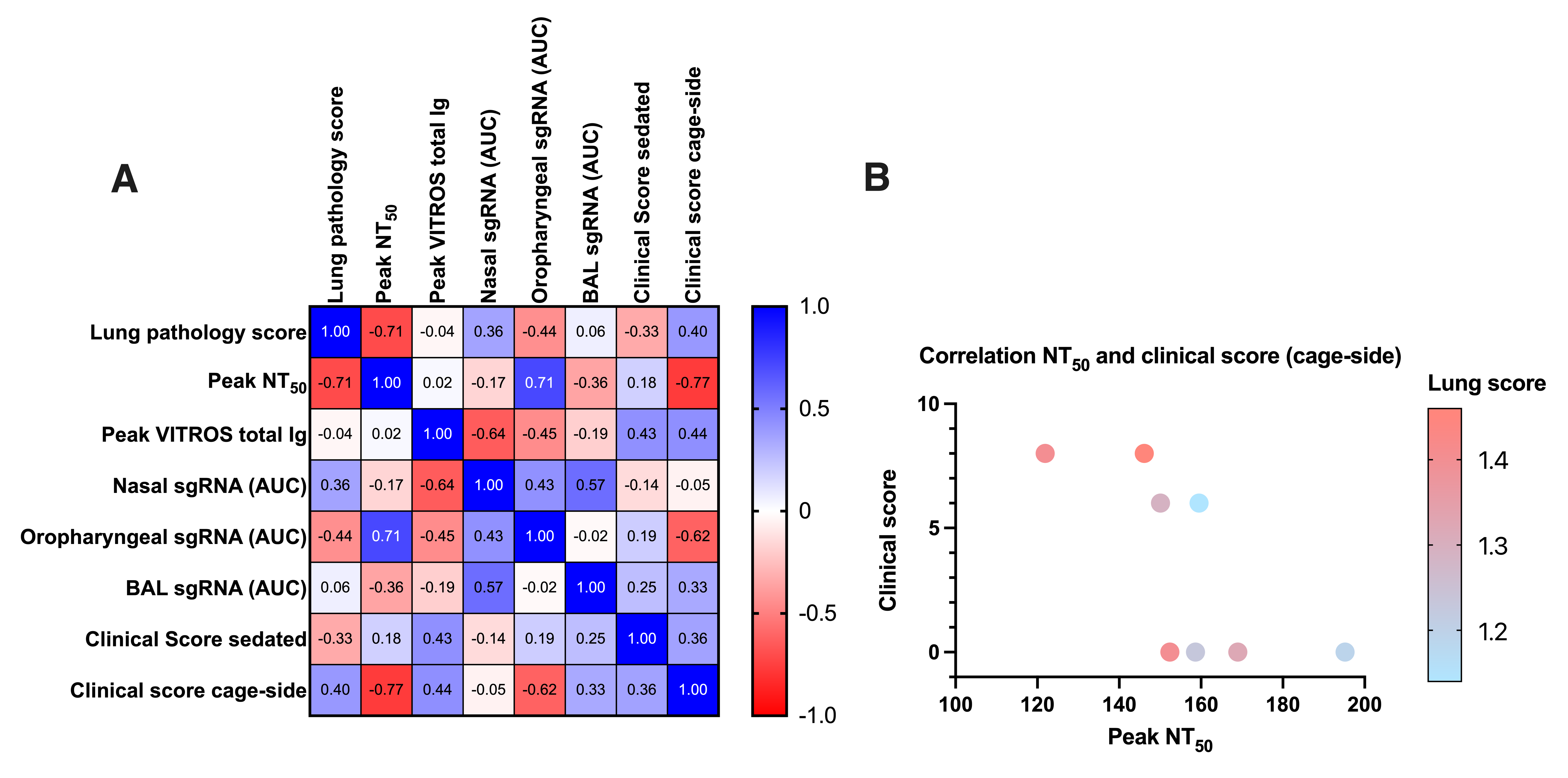

Supplement: S9 Fig — Multivariate analysis was performed on the 8 CCP-treated animals only. (A). Spearman r correlation matrix in heatmap format. For this analysis, the markers used are the same ones as in Fig 8. (B) Correlation between neutralizing antibody peak NT50 values and clinical scores based on cage-side observations (Spearman r = -0.77; p = 0.04). (TIFF) [file ppat.1009925.s009.tiff]

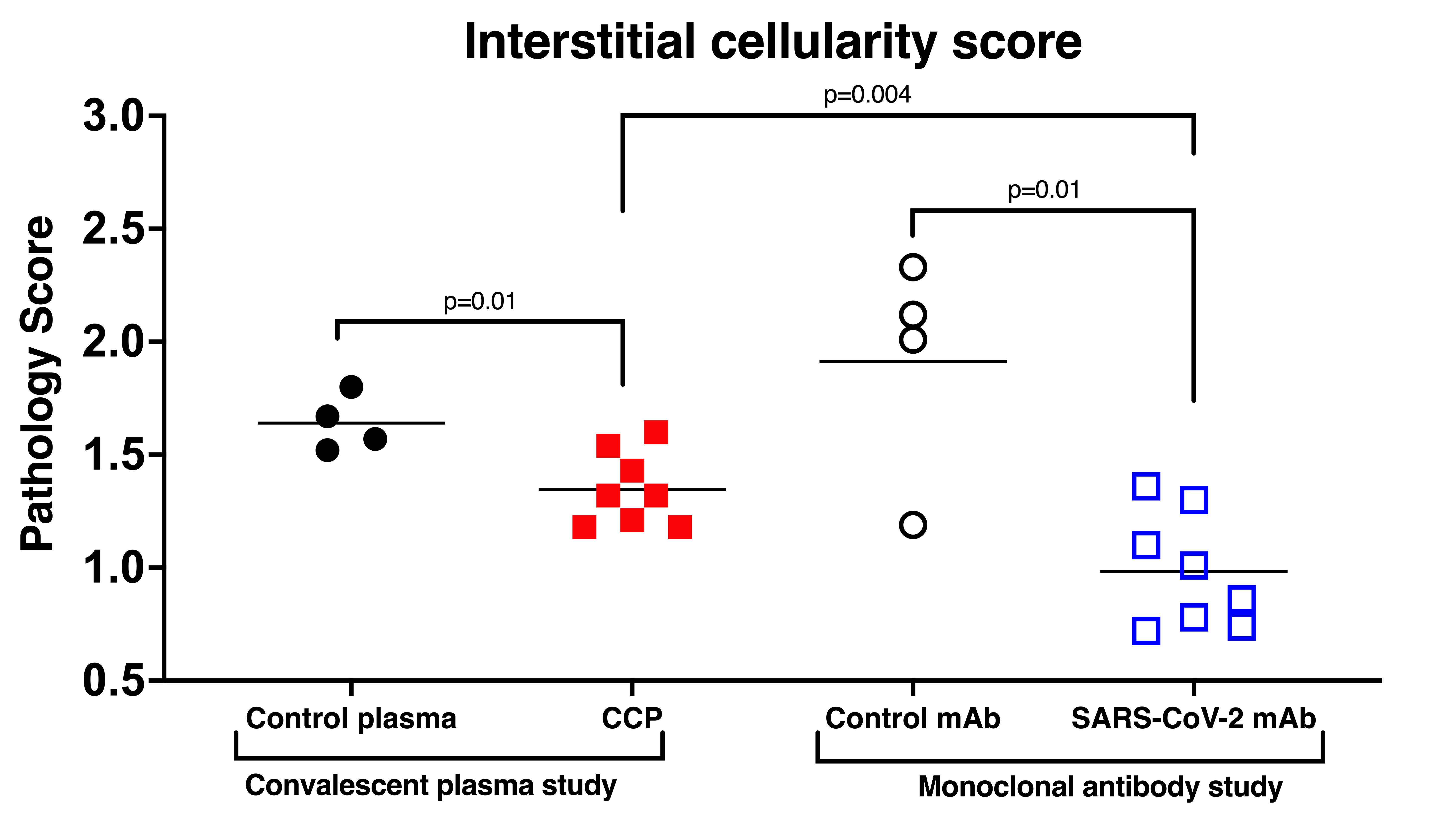

Supplement: S10 Fig — In an earlier study that used the same experimental procedures, we demonstrated that a combination of 2 potent anti-SARS-CoV-2 monoclonal antibodies (mAb; C135-LS and C144-LS) administered one day after virus inoculation reduced lung interstitial cellularity scores in 8 treated animals comparison to 4 animals treated with a control mAb [33]. Comparison of the CCP-treated and SARS-CoV-2 mAb-treated groups demonstrated that mAbs are more effective than CCP in reducing interstitial cellularity scores (p = 0.004, unpaired t test). Because in the monoclonal antibody study, scores were based on 3 lung lobes, the data of the CCP study presented in this figure are tabulated based on those same 3 lung lobes; using the data of all 7 lung lobes on the current CCP study (presented in Fig 7) resulted in the same conclusions. (TIFF) [file ppat.1009925.s010.tiff]
