# Supplementary material for: Early post-infection treatment of SARS-CoV-2 infected macaques with human convalescent plasma with high neutralizing activity had no antiviral effects but moderately reduced lung inflammation
Source: PLoS Pathog. 2022 Apr 20;18(4):e1009925. doi: 10.1371/journal.ppat.1009925 (PMC9060337; doi:10.1371/journal.ppat.1009925)
Supplement: S1 Table — Due to limited amount available of the plasma with the highest titer (W084520000915), a maximal amount of this highest-titer plasma was used and mixed with the 2 other units at a ratio of 60:20:20 in order to administer the maximum absolute amount of convalescent plasma-derived neutralizing antibodies to the animals. 1NT50 and NT80 titers determined by RVPN assay. 2Top percentile values of individual plasma units and the pooled CCP are based on 223 convalescent plasma samples with median NT50 titer of 784. 3Signal to cut-off ratio values on VITROS Total Ig assay (which measures anti-spike IgG, IgM and IgA). A value of ≥1 is considered reactive. (DOCX) [file ppat.1009925.s011.docx]

**S1 Table. Preparation of pooled COVID convalescent plasma.**

| **Truncated DIN** | **% of total pool** | **NT_50_**^1^ | **Top percentile based on NT_50_^2^** | **NT_80_**^1^ | **VITROS S/CO^3^** |
| --- | --- | --- | --- | --- | --- |
| W084520000915 | 60% | 18,922 | 1% | 2,313 | 736 |
| W041020069696 | 20% | 1,135 | 40% | 541 | 345 |
| W041120015179 | 20% | 1,350 | 35% | 454 | 506 |
| **Pooled CCP** | **100%** | **3,003** | **20%** | **1,113** | **684** |

Due to limited amount available of the plasma with the highest titer (W084520000915), a maximal amount of this highest-titer plasma was used and mixed with the 2 other units at a ratio of 60:20:20 in order to administer the maximum absolute amount of convalescent plasma-derived neutralizing antibodies to the animals**.**

^1^NT_50_ and NT_80_ titers determined by RVPN assay.

^2^Top percentile values of individual plasma units and the pooled CCP are based on 223 convalescent plasma samples with median NT_50_ titer of 784.

^3^Signal to cut-off ratio values on VITROS Total Ig assay (which measures anti-spike IgG, IgM and IgA). A value of ≥1 is considered reactive.
