# Supplementary material for: Early post-infection treatment of SARS-CoV-2 infected macaques with human convalescent plasma with high neutralizing activity had no antiviral effects but moderately reduced lung inflammation
Source: PLoS Pathog. 2022 Apr 20;18(4):e1009925. doi: 10.1371/journal.ppat.1009925 (PMC9060337; doi:10.1371/journal.ppat.1009925)
Supplement: S2 Table — Animals were inoculated with SARS-CoV-2 on day 0, and control or convalescent plasma was infused on day 1. 50% and 80% neutralization titers (NT50 and NT80) in serum were measured by a RVPN assay. Total Ig against spike protein was determined by the VITROS assay. Green shading indicates values above the cut-off of the respective assay. (DOCX) [file ppat.1009925.s012.docx]

**S2 Table**. **SARS-CoV-2 neutralizing and anti-spike antibodies in serum of macaques.**

Animals were inoculated with SARS-CoV-2 on day 0, and control or convalescent plasma was infused on day 1. 50% and 80% neutralization titers (NT_50_ and NT_80_) in serum were measured by a RVPN assay. Total Ig against spike protein was determined by the VITROS assay. Green shading indicates values above the cut-off of the respective assay.
