# Supplementary material for: Early post-infection treatment of SARS-CoV-2 infected macaques with human convalescent plasma with high neutralizing activity had no antiviral effects but moderately reduced lung inflammation
Source: PLoS Pathog. 2022 Apr 20;18(4):e1009925. doi: 10.1371/journal.ppat.1009925 (PMC9060337; doi:10.1371/journal.ppat.1009925)
Supplement: S3 Table — All thorax radiographs were scored blinded by a veterinary radiologist, with scores of 0 to 3 assigned to each of the 7 lung lobes. For each time point, the total score of all lung lobes was tabulated. Thus, the maximum score per time point is 21. (DOCX) [file ppat.1009925.s013.docx]

**S3 Table. Summary of radiological scoring.**

All thorax radiographs were scored blinded by a veterinary radiologist, with scores of 0 to 3 assigned to each of the 7 lung lobes. For each time point, the total score of all lung lobes was tabulated. Thus, the maximum score per time point is 21.
