# Supplementary material for: Early post-infection treatment of SARS-CoV-2 infected macaques with human convalescent plasma with high neutralizing activity had no antiviral effects but moderately reduced lung inflammation
Source: PLoS Pathog. 2022 Apr 20;18(4):e1009925. doi: 10.1371/journal.ppat.1009925 (PMC9060337; doi:10.1371/journal.ppat.1009925)
Supplement: S4 Table — (DOCX) [file ppat.1009925.s014.docx]

**S4 Table. Animal demographics**

| **Group** | **Animal ID** | **Sex** | **Age at time of inoculation (months)** | **Body weight at time of inoculation (kg)** |
| --- | --- | --- | --- | --- |
| Control plasma | Co-1 | M | 195 | 12.81 |
| “ | Co-2 | F | 169 | 9.31 |
| “ | Co-3 | M | 167 | 8.34 |
| “ | Co-4 | F | 110 | 6.09 |
|  |  |  |  |  |
| Convalescent plasma | CCP-1 | F | 196 | 10.05 |
| “ | CCP-2 | M | 166 | 7.99 |
| “ | CCP-3 | M | 163 | 9.79 |
| “ | CCP-4 | F | 137 | 6.70 |
| “ | CCP-5 | M | 135 | 10.22 |
| “ | CCP-6 | F | 112 | 6.01 |
| “ | CCP-7 | F | 111 | 5.82 |
| “ | CCP-8 | M | 110 | 6.22 |
