# Supplementary material for: Early post-infection treatment of SARS-CoV-2 infected macaques with human convalescent plasma with high neutralizing activity had no antiviral effects but moderately reduced lung inflammation
Source: PLoS Pathog. 2022 Apr 20;18(4):e1009925. doi: 10.1371/journal.ppat.1009925 (PMC9060337; doi:10.1371/journal.ppat.1009925)
Supplement: S5 Table — (DOCX) [file ppat.1009925.s015.docx]

**S5 Table. Flow cytometry antibody and reagents.**

| **S. No** | **Reagents** | **Source** | **Identifier** |
| --- | --- | --- | --- |
| 1. | AF488 anti-human Ki-67 (Clone B56) | BD Biosciences | Cat#558616 |
| 2. | AF700 anti-human CD14 (Clone MSE2) | BD Biosciences | Cat# 301822 |
| 3. | AF700 anti-human CD3 (Clone SP34-2) | BD Biosciences | Cat# 557917 |
| 4. | APC anti-human CD66 (Clone TET2) | Miltenyi Biotec | Order#130-118-539 |
| 5. | APC-Cy7 anti-human CD3 (Clone SP34-2) | BD Biosciences | Cat#557757 |
| 6. | APC-Cy7 anti-human CD20 (Clone 2H7) | BioLegend | Cat#302314 |
| 7. | APC-Cy7 anti-human live/dead | invitrogen | Ref#L34976A |
| 8. | BV421 anti-human CD123 (Clone 7G3) | invitrogen | Ref#48-1238-42 |
| 9. | BV605 anti-human CD16 (Clone 3G8) | BioLegend | Cat#302040 |
| 10. | BV650 anti-human CD4 (Clone L200) | BD Biosciences | Cat# 563737 |
| 11. | BV786 anti-human HLA-DR (Clone L243) | BioLegend | Cat#307642 |
| 12. | BUV737 anti-human CD95 (Clone DX2) | BD Biosciences | Cat# 564710 |
| 13. | BUV805 anti-human CD8 (Clone SK1) | BD Biosciences | Cat#612889 |
| 14. | PECF594 anti-human CD28 (Clone CD28.2) | BioLegend | Cat# 302942 |
| 15. | PECy7 anti-human CD11c (Clone 3.9) | invitrogen | Ref#25-0116-42 |
| 16. | PECy7 anti-human PD1 (Clone EH12.2H8) | BioLegend | Cat# 329918 |
| 17. | FACS lyse | BD Biosciences | Cat#349202 |
| 18. | FoxP3/ Transcription Factor Staining Buffer set | invitrogen | Cat#00-5523 |
| 19. | Brilliant stain buffer | BD Biosciences | Cat#563794 |
